# Feeding behaviour patterns in relation to body weight and gait in broilers

**DOI:** 10.1016/j.psj.2025.105103

**Published:** 2025-03-27

**Authors:** Malou van der Sluis, Britt de Klerk, István Fodor, Esther D. Ellen

**Affiliations:** a Animal Breeding and Genomics, Wageningen University & Research, 6700 AH, Wageningen, the Netherlands; b Cobb Europe, Boxmeer, the Netherlands

**Keywords:** Feeder visits, Chickens, RFID, Hock burn, Footpad dermatitis

## Abstract

Collecting data on broiler behaviour patterns in group-housed settings can be challenging, as broilers are difficult to recognize individually. However, broiler behaviour patterns can be valuable for assessing health, welfare and performance. Here, individual feeding patterns of group-housed broilers were studied. Data on feeder visits of 58 randomly selected and subsequently RFID-tagged broilers were used, that were housed in a group of approximately 800 birds in a 45 m^2^ pen. Feed and water were provided *ad libitum*, with a total of 16 feeders available to the broilers. All feeders except one (due to farm equipment blocking the access) were fitted with an RFID antenna to detect presence of the broilers at the feeders. The number of feeder visits and feeding durations at the individual level during the period from 20 to 30 d old were examined. In addition, birds’ individual body weights (g) were determined at 14, 21, 27 and 35 d of age, and at 21, 27 and 35 d of age gait scores were determined by experienced observers. The feeders used within the pen (i.e., whether feeding occurred in one area or was spread out across the pen) varied between individuals, and for some birds also changed with age. Furthermore, with higher body weights at 14 d of age, the number of different feeders visited in a day was lower (estimate = -0.011, *P* = 0.002) and the mean feeding bout duration was higher (estimate = 0.130, *P* = 0.007). In contrast, larger body weight gain between 14 and 35 d of age was related to more different feeders visited within a day (estimate = 0.028, *P* = 0.005) and shorter feeding bout durations (estimate = -0.390, *P* = 0.005). No relationships of feeding descriptors with gait classification were observed. Overall, feeding patterns vary between individual broilers and, given the observed relationships between feeding patterns and weight gain, feeding patterns have potential to be informative for broiler growth in research and commercial conditions.

## Introduction

Improving the welfare of farm animals is considered important in Europe (European Commission, 2023). For broilers, or meat chickens, main welfare concerns include impaired walking ability (Knowles et al., 2008) and impaired health (de Jong, 2020). Possibly, assessing the behaviour of broilers can provide insight into their health, welfare and performance. However, broilers are commonly kept in groups of thousands of birds. Collecting data on broiler behaviour patterns can be challenging in such group-housed settings, as broilers are difficult to recognize and monitor individually due to their highly similar appearance. However, body-worn sensor technologies, such as radio frequency identification (**RFID**) tracking, ultra-wideband tracking or the use of accelerometers, can help to collect data at the individual level in poultry (Ellen et al., 2019; van der Sluis et al., 2019, 2020; Baxter and O'Connell, 2023; Alindekon et al., 2023). Also remote, vision-based techniques (using cameras) can provide insight into bird behaviour, although individual identification remains challenging (Ellen et al., 2019).

One important behavioural trait in broilers is feeding, as it may provide insight into several health, welfare and performance aspects. For example, feed intake is positively related to body weight (**BW**) gain in broilers (Sung and Adeola, 2022) and thus to their productive performance. Furthermore, both BW and growth rate have been shown to be linked to broiler gait (Kestin et al., 2001). Feeding patterns may change depending on a broiler's health or welfare status. Reductions in feed intake may be indicative of disease in animals (Gregory, 1998) and birds with gait problems may show altered behaviour and activity levels (e.g., Riber et al., 2021a; van der Sluis et al., 2021) which one might expect to also impact feeding patterns, for example by visiting the feeders less often but for longer meals. Overall, feeding behaviour patterns over time appear to be highly relevant to monitor in broilers, due to the insights these may provide into growth, health and walking ability.

In this study, we examined broiler feeding behaviour patterns in two main ways: 1) a descriptive overview of individual differences in temporal and spatial feeder visit patterns, and 2) a more detailed statistical analysis of three components of feeding behaviour (i.e., the total number of feeder visits per day, the mean feeding bout duration per visit, and the number of different feeders visited per day) in relation to age, BW and leg health. The results of this study can contribute to a better understanding of broiler feeding patterns and the relationships with health, welfare and performance.

## Materials and methods

### Ethical statement

Data were collected under control of Cobb Europe (Boxmeer, the Netherlands). Cobb Europe complies with the Dutch legislation on animal welfare. This study is not considered to be an animal experiment under the Law on Animal Experiments, as confirmed by the local Animal Welfare Body (July 11, 2022, Lelystad, the Netherlands).

### Housing and birds

**Fig. 1:**
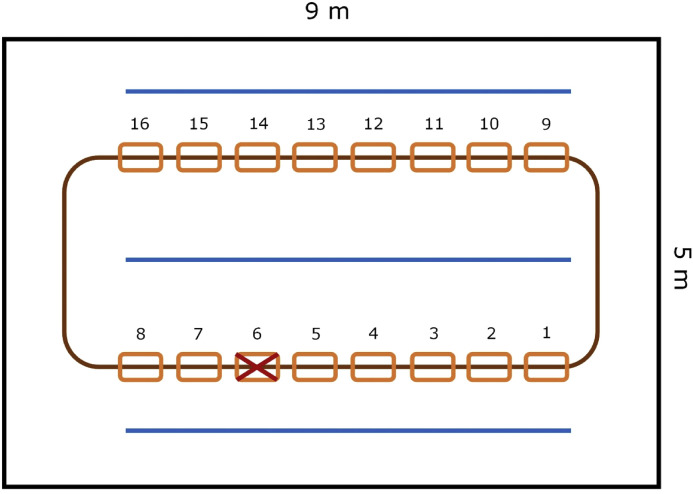
Schematic layout of the broilers’ pen. Blue lines represent the drinker lines, the brown line represents the feeder line, with orange rectangles indicating each of the feeders (numbered). One feeder could not be fitted with an RFID antenna, as indicated with a cross in this figure.

All data were collected on a broiler farm in the Netherlands. A total of approximately 800 broilers of a fast-growing cross were housed in a pen with a size of approximately 9 by 5 m (45 m^2^). In this pen, two feeder lines, with a total of 16 feeders, and three drinking lines were present for *ad libitum* access to feed and water (Fig. 1). Wood shavings were provided as bedding. Commercial lighting and temperature schedules were used and the birds were all vaccinated according to common practice (Cobb, 2018). At 14 d of age, a total of 73 broilers (40 males, 33 females) were tagged with a high frequency (**HF**) RFID tag in the wing for identification and tracking. These birds were randomly selected from different areas of the pen and subsequently approximately equal numbers of males and females were included. Only a small subset of the birds in the pen were tagged, as the data were originally collected for a larger broiler tracking pilot study that did not focus specifically on feeding behaviour. However, given that the collected data included individual-level tracking of presence at the feeders, with a total of almost 36,000 feeder visits for 58 individuals across 11 days (see further on for how feeder visits were defined and how this final sample was obtained), it allowed us to study individual variation in feeding behaviour of broilers.

### Tracking feeder visits

**Fig. 2:**
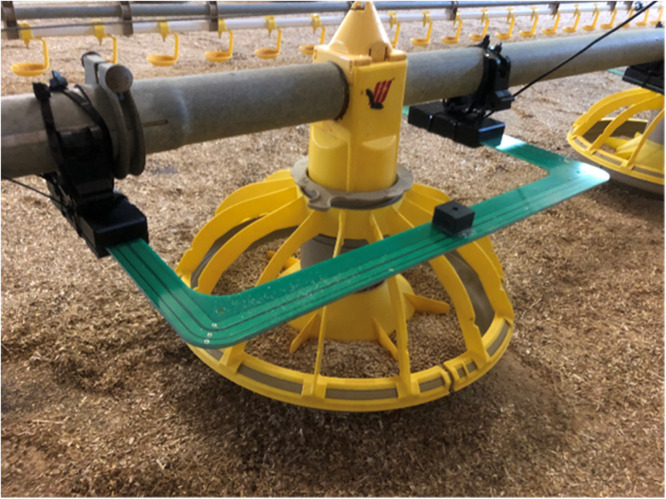
Feeder fitted with an HF RFID antenna.

Of the 16 feeders in the pen, we fitted 15 with an HF RFID antenna from Dorset Identification (Dorset Identification B.V., Aalten, the Netherlands) (Fig. 2). One feeder could not be fitted with an antenna due to a metal structure on the feeder line blocking access for the antenna (see Fig. 1). The 15 antennas were connected to one of two readers, and detected the RFID tags of the birds present within the antenna range (up to a few cm distance from the antenna itself) at 1 Hz frequency. Using custom-made software (freaquent froschelectronics GmbH, Graz, Austria), a log file was stored with the bird ID (i.e., unique RFID tag code), the location of the antenna (i.e., the pre-assigned antenna number) and the date and time of registration. Based on this log file, the time and location of each feeder visit of each RFID-tagged bird could be determined. RFID data were available and complete for the period from 20 to 30 d of age (due to technical problems, data were incomplete for the preceding days), although it is important to note that this period included two moments (at 21 and 27 d of age) during which the birds were taken from the pen for a short period of time to assess BW and leg health.

Using the information from the log file, feeder visits and feeding durations could be determined. A threshold for feeder visits at the same feeder was defined, as it might happen that a bird was at the feeder but was not detected at the associated antenna for a couple of seconds. This could for example happen when a bird changed position at the same feeder or lay down for a couple of seconds. Here, gaps of over 24 s in the RFID records were considered as true gaps in feeding and shorter gaps were considered as the same feeding bout. As our RFID system automatically continued reporting a bird at the antenna where it was last detected for six seconds after its last actual detection, this resulted in a total feeding duration gap threshold of 30 s. Based on the resulting feeding bout information, we derived for each bird and day the spatial and temporal pattern of feeder visits, as well as three feeding behaviour descriptors: 1) the number of feeder visits in a day (**NFV**), 2) the mean feeding bout duration per visit (**MFBD**), and 3) the number of different feeders visited in a day (**NDF**; i.e. how many of the 15 different feeders were visited in a day).

### Body weight and leg health scoring

The birds’ individual BWs were determined at 14, 21, 27 and 35 d of age, using a scale with one-gram precision (Table 1). Moreover, after weighing on d 21, 27 and 35, the birds’ hock burn (**HB**) scores and foot pad dermatitis (**FPD**) scores were determined by an experienced observer (Table 1). Both HB and FPD were scored on a scale from zero to five, with separate scores for the left and right hock or foot. For both the HB and the FPD scores, the scores of the left and the right leg were summed up, to obtain a single HB and FPD score per animal per age, respectively. The walking ability (i.e., gait score) of the birds was scored on a scale from zero to five (described earlier by van der Sluis et al. (2021)), by observing the birds walk when placed back in the home pen after weighing. Due to the small sample size, birds were subsequently categorized into two gait classes, with ‘good gait’ (**GG**) as gait scores 2 or lower and ‘suboptimal gait’ (**SG**) as gait scores 3 or higher (Table 1). In all subsequent analyses, the gait score category recorded at 27 d of age was used as a representation of birds’ walking ability, as birds’ gait scores changed over time (Table 1) and the gait score at 27 d of age overlapped best with the recording period of the feeder visits (i.e., 20 to 30 d of age). Due to misidentification (*n* = 13) and mortality of birds (*n* = 2), complete data were available for a total of 58 birds (Table 1). As there were strong correlations between the BWs at different ages for these birds (i.e., at 14, 21, 27 and 35 d of age; ranging from 0.42 (for 14 versus 35 d of age) to 0.92 (for 21 versus 27 d of age), all *p* < 0.01), only the start BW (14 d of age) and the BW gain (i.e., the calculated difference in BW between d 14 and 35, divided by the number of days in between) were included in further analyses.

**Table 1:** Mean body weights and leg health scores (±SD) of the birds at different ages in the trial (*n* = 58).

Age (days)	Body weight (grams)	Hock burn score	Footpad dermatitis score	Gait score	Gait classification
14	596 (±52)	NA	NA	NA	NA
21	1137 (±132)	1.4 (±0.8)	1.0 (±0.1)	1.6 (±0.6)	*n* = 56 good gait *n* = 2 suboptimal gait
27	1701 (±237)	2.2 (±1.8)	1.3 (±0.8)	2.2 (±0.9)	*n* = 41 good gait *n* = 17 suboptimal gait
35	2515 (±400)	3.6 (±2.2)	2.0 (±1.9)	2.6 (±0.8)	*n* = 32 good gait *n* = 26 suboptimal gait

### Statistical analyses

For all statistics, R version 4.1.0 was used (R Core Team, 2021). First, the spatial and temporal patterns of feeder visits were examined descriptively. To this end, the presence at feeders was plotted over time, per bird and day, using the ggplot2 package (Wickham, 2016). Second, the three feeding behaviour descriptors (NFV, MFBD and NDF) were examined in more detail. Extreme outliers for NFV, MFBD and NDF were identified using a threshold of four times the standard deviation and these individual observations per day were excluded from the analyses (one for NFV, four for MFBD). To study how BW, BW gain and gait were related to feeding behaviour while accounting for other potentially influential factors (i.e., sex and age of the birds, as these may also affect feeding behaviour), linear mixed-effects models with sum-to-zero contrasts were implemented, using the lme4 (Bates et al., 2015) and lmerTest (Kuznetsova et al., 2017) packages. For this analysis, a total of 632 observations for 58 animals were used. All predictors deemed biologically relevant – age (in days), sex (male versus female), gait classification (GG versus SG; at 27 d of age), start BW (in grams, at 14 d of age), BW gain (in grams per day, between 14 and 35 d of age) – and their two-way interactions were included in the model as fixed effects, and bird as a random effect. Interactions and main effects that were not statistically significant (*P* > 0.05) were removed using backward selection. This resulted in the following models for NFV, MFBD and NDF, respectively:

(1) $$NFV_{ij}=\mu +\;\beta_{1}\left(Age_{ij}\right)+u_{j}+e_{ij}$$

(2) $$MFBD_{ij}=\mu +\;\beta_{1}\left(Age_{ij}\right)+\beta_{2}\left(SW_{j}\right)+\;\beta_{3}\left(WG_{j}\right)+u_{j}+e_{ij}$$

(3) $$NDF_{ij}=\mu +\;\beta_{1}\left(Age_{ij}\right)+\beta_{2}\left(SW_{j}\right)+\;\beta_{3}\left(WG_{j}\right)+u_{j}+e_{ij}$$

where μ is the overall mean, $Age_{ij}$ is the *i*^th^ day of age (*i* = 20 to 30) of bird *j*, $SW_{j}$ is the start BW of bird *j* at 14 d of age, $WG_{j}$ is the BW gain of bird *j* between 14 and 35 d of age, $\beta_{1}$, $\beta_{2}$, and $\beta_{3}$ are the respective regression coefficients, $u_{j}$ is the random intercept for the *j^th^* bird, and $e_{ij}$ is the residual term. No random slopes were added, because in all models this resulted in very high correlations between the slopes and intercepts, ranging from −0.94 to −0.97. The performance package (Lüdecke et al., 2021) was used to visually check model assumptions. No obvious deviations from normality or homoscedasticity were observed upon visual inspection of the residuals of the models. Reported P-values for the model estimates were obtained using the lmerTest package (Kuznetsova et al., 2017). The MuMIn package (Barton, 2020) was used to determine the R^2^ values for the models. The ggplot2 package (Wickham, 2016) was used to make the visualizations. The level of statistical significance was set at 0.05.

## Results

### Spatial and temporal feeding patterns

**Fig. 3:**
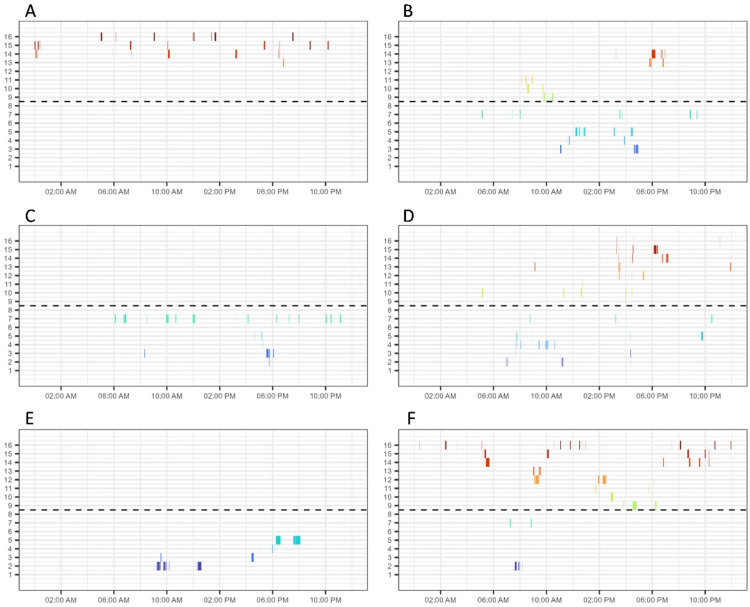
Contrasting examples of birds remaining more in one location within a day (left, 3 different birds (panels A, C, E)) and birds using feeders throughout the pen (right, 3 different birds (panels B, D, F)). On the y-axis the antenna (or feeder) number is shown (see Fig. 1 for location reference) and on the x-axis the time within the day is shown. The dashed line represents the boundary between the virtual two halves of the pen. Different colours are used to indicate different feeders for easy visual distinction.

**Fig. 4:**
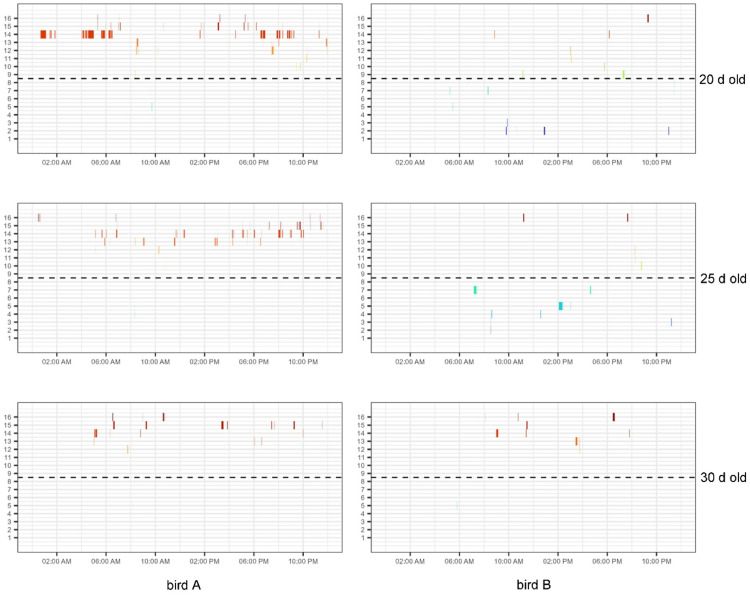
Contrasting examples of a bird showing a similar pattern across days (left; bird A) and a bird showing different patterns across days (right; bird B). Top row: 20 d old; middle row: 25 d old; bottom row: 30 d old. On the y-axis the antenna (or feeder) number is shown (see Fig. 1 for location reference) and on the x-axis the time within the day is shown. The dashed line represents the boundary between the virtual two halves of the pen. Different colours are used to indicate different feeders for easy visual distinction.

When looking at the overall feeder visits across the tagged birds, we observed relatively few visits to feeders 1 and 8, which were placed at the far ends of the feeder line on one side of the pen (data not shown; see Fig. 1 for location reference). However, individual birds showed variation in their spatial and temporal feeding patterns. For example, some birds remained more in one location within a day, whereas others used feeders throughout the pen (see Fig. 3 with several manually selected contrasting examples). Furthermore, some birds showed a few longer-lasting visits (e.g., Fig. 3**E**) whereas other birds showed more frequent short-lasting visits (e.g., Fig. 3**A**). Some birds were quite consistent in the feeders they visited on different days, whereas other birds showed more variation over time (see Fig. 4 with two manually selected contrasting examples).

### Feeding behaviour descriptors

**Fig. 5:**
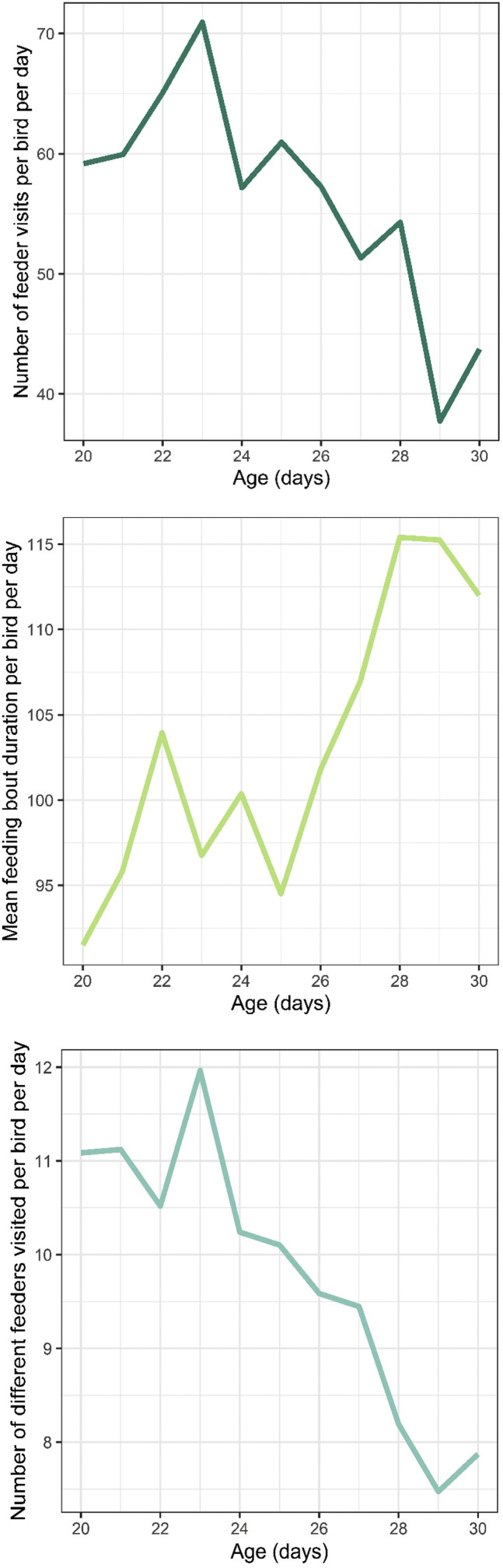
Mean values of the feeding descriptors over time. Top) Number of feeder visits per bird per day, middle) mean feeding bout duration per bird per day (in seconds), bottom) number of different feeders visited per bird per day.

Fig. 5 shows the mean values for the three feeding behaviour descriptors over time. To study feeding behaviour in more detail, we analysed the number of feeder visits, mean feeding bout duration, and the number of different feeders visited (Tables 2**–**4). In all models, sex and gait score category were not statistically significant predictors and were already excluded in the model selection stage. Correlations between bird characteristics (i.e., sex, gait classification and BWs) and feeding behaviour descriptors are presented in **Supplementary data 1**.

**Table 2:** Results of the linear mixed-effects model for the number of feeder visits. The initial model included age, sex, gait classification, start body weight and body weight gain as fixed effects, of which only age remained after backward selection. Bird identity was included as a random effect.

Number of feeder visits					
***Fixed effects***					
Factor	F-value	Pr(>*F*)	Estimate	SE	Pr(>|t|)
Intercept			111.411	6.427	<0.001
Age (d)	83.102	<0.001	−2.212	0.243	<0.001
***Random effects***					
Factor	Variance	SD			
ID intercept	232.4	15.24			
Residual	368.3	19.19			

The model for NFV explained 7.48 % of the overall variance when only fixed effects were included and 43.28 % when the random bird effect was included too. The NFV decreased as the birds grew older (Table 2).

The model for MFBD explained 9.04 % of the overall variance when only fixed effects were included and 27.14 % when the random bird effect was included. Birds with higher start BW at 14 d of age had longer feeding bouts, and bouts became shorter with higher BW gain. Furthermore, MFBD increased with age (Table 3).

**Table 3:** Results of the linear mixed-effects model for the mean feeding bout duration (in seconds). The initial model included age, sex, gait classification, start body weight and body weight gain as fixed effects, of which age, start body weight and body weight gain remained after backward selection. Bird identity was included as a random effect.

Mean feeding bout duration					
***Fixed effects***					
Factor	F-value	Pr(>*F*)	Estimate	SE	Pr(>|t|)
Intercept			6.198	28.638	0.829
Age (d)	33.411	<0.001	2.192	0.379	<0.001
Start weight (g)	7.766	0.007	0.130	0.047	0.007
Weight gain (g/d)	8.416	0.005	−0.390	0.134	0.005
***Random effects***					
Factor	Variance	SD			
ID intercept	223.5	14.95			
Residual	900.0	30.00			

The model for NDF explained 25.20 % of the overall variance when only fixed effects were included and 40.26 % when the random bird effect was also included. Birds with higher start BW at 14 d of age visited less different feeders within the pen, whereas birds showing higher BW gain visited more different feeders within a day. Birds visited a lower number of different feeders within a day as they grew older (Table 4).

**Table 4:** Results of the linear mixed-effects model for the number of different feeders visited. The initial model included age, sex, gait classification, start body weight and body weight gain as fixed effects, of which age, start body weight and body weight gain remained after backward selection. Bird identity was included as a random effect.

Number of different feeders visited					
***Fixed effects***					
Factor	F-value	Pr(>*F*)	Estimate	SE	Pr(>|t|)
Intercept			23.702	2.054	<0.001
Age (d)	213.406	<0.001	−0.396	0.027	<0.001
Start weight (g)	10.768	0.002	−0.011	0.003	0.002
Weight gain (g/d)	8.325	0.005	0.028	0.010	0.005
***Random effects***					
Factor	Variance	SD			
ID intercept	1.155	1.075			
Residual	4.586	2.141			

The results for all three feeding behaviour descriptors are visually summarized in Table 5. It appears that NDF and MFBD tend to show effects in the opposite direction: with increasing age or start BW, MFBD increases while NDF decreases, while with larger BW gain MFBD decreases and NDF increases.

**Table 5:** Visual summary of the results for the three feeding behaviour descriptors. NFV = number of feeder visits; MFBD = mean feeding bout duration; NDF = number of different feeders visited. Arrows indicate a decrease (↓) or increase (↑) in the feeding descriptor (i.e., in number of visits, visit duration or number of different feeders visited) in response to the effects listed in the first column, based on the outcomes of the linear mixed effects models.

	NFV	MFBD	NDF
Increasing age	↓	↑	↓
Increasing start body weight	**-**	↑	↓
Larger body weight gain^1^	**-**	↓	↑

1 i.e., the calculated difference in body weight between 14 and 35 d of age, divided by the number of days in between.

## Discussion

In this study, feeding patterns of group-housed broilers were investigated. We provided a descriptive overview of individual differences in temporal and spatial feeder visit patterns, and assessed the effects of BW (gain) and gait on three components of daily feeding behaviour (NFV, MFBD and NDF). Even though the sample size was small due to the data originally having been collected for a different purpose, we observed individual variation in the location of feeders visited and in the consistency of the spatial patterns across days. Furthermore, we observed that BW (gain) is linked to differences in feeding behaviour. Birds with a higher BW at 14 d of age visited fewer different feeders in a day, but had longer mean feeding bout durations, than birds with lower BW at 14 d of age. Birds with a larger BW gain between 14 and 35 d of age visited more different feeders in a day, but had shorter mean feeding bout durations, than birds with a smaller BW gain across this period. We observed no relationship between gait score classification and feeding behaviour. Overall, the results of this study suggest that data on feeding behaviour can provide insight into BW (gain), but are – in their current form – not informative for walking ability in broilers.

### Spatial and temporal feeding patterns

In this research, we observed differences in spatial and temporal feeding patterns of broilers and in how consistent birds were in their patterns. It must, however, be noted that these differences were observed based on visual (manual) inspection of the feeder visits over time and were not statistically assessed. Nonetheless, similar observations, but regarding use of the area in a commercial house of 28,000 birds, were made by Baxter and O'Connell (2023). They used an ultra-wideband tracking system and showed that while some broilers spent most time within a 10 m range from where they were originally found, others visited at least 90 % of the house. Birds’ feeding location patterns may be linked to their environmental preferences, which may also differ between individual birds. Light conditions can have an impact, as it has been shown, in a choice trial with three light intensities (5, 10 or 20 lux), that most birds were present and most feed was consumed in the 20 lux light condition (Raccoursier et al., 2019). Furthermore, the level of disturbance in different areas may affect where birds prefer to feed. Birds appear to prefer areas with fewer disturbances by other birds, for example caused by traffic between the feeders and drinkers (Li et al., 2020), and tend to perform eating behaviour at larger distances from their nearest neighbours than when performing other behaviours such as preening, lying down or sitting (Buijs et al., 2011). Another factor that might have an impact on feeder preference is the proximity of drinkers. It has been shown that, at low densities, birds tend to prefer to stay close to feeders and drinkers (Arnould and Faure, 2004), so feeders close to drinkers might be preferred. However, in the current study the distance between each feeder and its nearest drinker was equal (see Fig. 1), so this is unlikely to have impacted the feeding behaviour patterns observed in this study. Besides environmental factors, it might also be expected that birds with gait problems remain more in one area (and thus visit feeders only in a small area), as walking might be painful for birds with poor gait scores (Danbury et al., 2000), and that birds with higher BWs remain more in one area because of the sometimes observed lower locomotor activity of heavier birds (Tickle et al., 2018; van der Sluis et al., 2019). However, Baxter and O'Connell (2023) observed no clear relationship between movement patterns and gait score or BW in their study on individual broiler movement patterns. Besides the here-discussed temporal and spatial aspects of feeding behaviour, we observed individual variation in the length and number of feeding bouts. The relationships between on the one hand the number of feeder visits, the mean feeding bout duration and the number of different feeders visited per day and on the other hand the birds’ BW (change) and gait are discussed below.

### Feeding behaviour descriptors

Birds with a higher body weight at 14 d of age showed a lower NDF and a higher MFBD, compared to birds with a lower body weight at 14 d of age. A lower NDF for birds with a higher BW at 14 d of age might be linked to the earlier-mentioned observations that birds with higher BW show lower (locomotor) activity levels (Tickle et al., 2018; van der Sluis et al., 2019). This might be linked to locomotion being energetically expensive in these heavy birds (Tickle et al., 2018). Possibly, as a consequence, heavier birds remain more in one location and thus visit fewer different feeders. Moreover, it has been indicated that birds with higher BW tend to have worse gait scores (Kestin et al., 2001; Riber et al., 2021b). Birds with poor gait scores might experience pain (Danbury et al., 2000) and show a reduction in activity (Weeks et al., 2000), potentially resulting in fewer different feeders being visited. However, we observed no statistically significant correlations between BW (gain) and gait classification (**Supplementary data 1**), although it must be noted that our sample size was limited. Regarding longer mean feeding bout durations, one might expect a lower total number of feeder visits when the mean feeding bout duration is longer. Yan et al. (2019) reported negative correlations between the number of feeder visits per day and the feeding duration per visit (*r* = −0.36) or the feed intake per visit (*r* = −0.95), in slow-growing N204 pure line yellow broilers from 57 to 77 d of age. Howie et al. (2011) also observed a negative phenotypic correlation between the number of meals per day and the meal duration (*r* = −0.11, *p* < 0.01) in four broiler lines. Also in the current study, we observed a negative correlation between NFV and MFBD (*r* = −0.33, *P* < 0.01; **Supplementary data 1**). Our observation that birds with a higher body weight at 14 d of age showed a higher MFBD contrasts with observations by Yan et al. (2019), who did not observe a statistically significant correlation between the start body weight (at 57 d of age) and the feeding duration per visit, but did observe a negative correlation between the start BW and the number of feeder visits (*r* = −0.21). In the current study, we did not observe a statistically significant relationship between start BW and NFV. Given that here NFV did not decrease with higher start BW, whereas the MFBD did increase, it might be that heavier birds consumed more feed or ate slower, but no data were available on feed intake here, so this remains to be investigated.

For a larger BW gain from 14 to 35 d of age, we observed opposite effects compared to higher start BW in terms of MFBD and NDF: with increasing BW gain, NDF increased and MFBD decreased. A possible explanation for this observation is that BW gain (from 14 to 35 d of age) might be negatively correlated with start BW (at 14 d of age), i.e., that birds with lower start BW would ‘catch up’ in their growth by a larger BW gain. However, in checking the model assumptions, no problems with correlations of the fixed effects were observed, and when further examining the relationship between BW gain and start BW a weak positive correlation was observed (*r* = 0.30, *p* < 0.001; **Supplementary data 1**). Therefore, a potential negative correlation between start BW and BW gain does not appear to explain the observations made in this study. Alternatively, growth might be indicative of good health, or at least the absence of poor health (e.g., not ill, not impaired by leg health problems), and therefore those birds with higher growth rates from 14 to 35 d of age might be healthier and subsequently move around the available area more and visit more different feeders in a day, with shorter average durations per feeder visit. Nonetheless, the results for BW at 14 d of age and BW gain across the period from 14 to 35 d of age appear to be somewhat contradictive. More research, including individual level feed intake and daily growth records (both of which were not available in this study), would be of great added value to elucidate the relationship between feeding patterns and BW (gain), especially as it has been suggested in literature that there might be a range of feeding strategies that may result in the same feed conversion ratio (Howie et al., 2011). Howie et al. (2011) studied genetic and phenotypic relationships between feeding behaviour and performance traits in four broiler lines. They observed, among other things, a positive phenotypic correlation between the number of meals per day and the average daily feed intake (averaged across the four genetic lines; *r* = 0.26, *p* < 0.01), as well as a strong positive genetic correlation between the average daily feed intake and feed conversion ratio (*r* = 0.91). When feed intake data are available, the relationship between feeding behaviour and BW (gain) can be examined in more detail.

Regarding gait scores, we observed no relationships with NFV, MFBD and NDF. Similarly, Baxter and O'Connell (2023) observed no clear relationship between gait score and space use (which might resemble NDF in the current study) or activity levels (which might relate to NFV in the current study). Broilers with the same gait scores show substantial individual variation in their activity levels and broilers with different gait scores show overlap in their activity levels (van der Sluis et al., 2021), and therefore individual variation might mask any potential effects of gait score on feeding behaviour patterns.

With increasing age, NFV and NDF decreased, while the mean feeding bout duration increased. These effects of increasing age may be linked to the decrease in overall locomotor activity as broilers age that has been reported in literature (Tickle et al., 2018; van der Sluis et al., 2019; Baxter and O'Connell, 2023), and is potentially linked to broilers’ increasing BW (Bokkers and Koene, 2003). It appears that, when the birds grow older, the decrease in NFV is compensated for by longer feeding bout durations, as is in line with the earlier discussed studies by Yan et al. (2019) and Howie et al. (2011).

### Towards practical implementation

The results of this study show that data on feeding behaviour can provide insight into BW (gain), but are – in their current form – not informative for walking ability in broilers. However, it is important to note that BW (gain) explained only a small part of the observed variation in the feeding descriptors (based on the R^2^ values of the models). Therefore, variation in feeding behaviour may be caused by more than BW aspects alone. What other aspects may also affect differences in feeding behaviour between individual broilers requires more research in the future. Nonetheless, keeping track of feeding behaviour may have added value in practice, to provide a first indication of performance in terms of BW gain, and to for example quickly detect feeder equipment malfunction (e.g., based on fewer or shorter visits to specific feeders) or keep track of deviations in feeding behaviour over time, which could be indicative of health problems or discomfort of the birds, using birds as their own reference (e.g., a bird suddenly visits the feeders much less often than on previous days). Although not tested in this study, these applications may contribute to quick detection and intervention, and hereby to improving broiler health and welfare in practice.

## Conclusion

Overall, this study showed that there is individual variation in feeding patterns, that is linked to differences in birds’ BW and BW gain. No relationship with gait was observed. More research, including individual level feed intake and daily growth records, would be of great added value to elucidate the relationship between feeding behaviour and BW (gain) and to assess how differences in feeding behaviour patterns relate to health and welfare in broilers. As yet, assessing broiler performance in terms of growth based on feeding behaviour data alone is challenging, as feeding behaviour is likely affected by more than just BW. Nonetheless, keeping track of feeding behaviour may have added value in practice, to provide a first indication of performance in terms of BW gain and to for example quickly detect feeder equipment malfunction or keep track of deviations in feeding behaviour over time, which could be indicative of health problems or discomfort of the birds, although these last two applications require validation in research.

## Funding

This study was supported by the Dutch Ministry of Economic Affairs (TKI Agri & Food project LWV21030) and the project partners Cobb Europe (Boxmeer, the Netherlands), Dorset Identification B.V. (Aalten, the Netherlands), and FarmResult (Wierden, the Netherlands).

## Disclosures

The authors declare that they have no known competing financial interests or personal relationships that could have appeared to influence the work reported in this paper.

## Appendix Supplementary materials

Image, application 1
