# Supplementary material for: Feeding behaviour patterns in relation to body weight and gait in broilers
Source: Poult Sci. 2025 Mar 27;104(6):105103. doi: 10.1016/j.psj.2025.105103 (PMC11997320; doi:10.1016/j.psj.2025.105103)
Supplement: Supplementary file 1 [file mmc1.docx]

**SUPPLEMENTARY DATA 1**

**Table S1** shows the correlations between bird characteristics (i.e., sex, gait classification and body weights) and feeding behaviour descriptors, averaged across days.

**Table S1: Correlations between bird and feeding behaviour descriptors, averaged across days. Correlations are given above the diagonal, P-values are given below the diagonal.** NFV = number of feeder visits; MFBD = mean feeding bout duration; NDF = number of different feeders visited.

|  | Sex | Start weight | Weight gain | Gait classification | NFV | MFBD | NDF |
| --- | --- | --- | --- | --- | --- | --- | --- |
| Sex |  | 0.41 | 0.39 | 0.26 | 0.09 | -0.05 | -0.04 |
| Start weight | <0.01 |  | 0.30 | 0.22 | -0.20 | 0.24 | -0.29 |
| Weight change | <0.01 | 0.02 |  | 0.05 | 0.10 | -0.26 | 0.23 |
| Gait classification | 0.05 | 0.10 | 0.71 |  | -0.16 | -0.03 | -0.25 |
| NFV | 0.51 | 0.13 | 0.44 | 0.23 |  | -0.33 | 0.40 |
| MFBD | 0.73 | 0.07 | 0.05 | 0.81 | 0.01 |  | -0.43 |
| NDF | 0.78 | 0.03 | 0.08 | 0.05 | <0.01 | <0.001 |  |
